# Supplementary material for: Mn2+ Luminescence in Ca9Zn1–xMnxNa(PO4)7 Solid Solution, 0 ≤ x ≤ 1
Source: Materials (Basel). 2023 Jun 14;16(12):4392. doi: 10.3390/ma16124392 (PMC10301442; doi:10.3390/ma16124392)
Supplement: Supplementary file 1 [file materials-16-04392-s001.zip › materials-2396453-supplementary.pdf]

materials

Supporting Information

Mn^{2+} Luminescence in $Ca_9Zn_{1-x}Mn_xNa(PO_4)_7$ Solid Solution, $0 \leq x \leq 1$

Eldar M. Gallyamov [1], Vladimir V. Titkov [1], Vladimir N. Lebedev [1], Sergey Y. Stefanovich [1], Bogdan I. Lazoryak [1] and Dina V. Deyneko [1,2]

[1] Department of Chemistry, Lomonosov Moscow State University, 119991 Moscow, Russia; e.m.gallyamov@gmail.com (E.M.G.); vlatitkov@yandex.ru (V.V.T.); vladimir.lebedev@chemistry.msu.ru (V.N.L.); s_stefanovich@mail.ru (S.Yu.S.); bilazoryak@gmail.com (B.I.L.); deynekomsu@gmail.com (D.V.D.)

[2] Laboratory of Arctic Mineralogy and Material Sciences, Kola Science Centre, Russian Academy of Sciences, 184209 Apatity, Russia

* Correspondence: s_stefanovich@mail.ru

Table S1. Crystallographic data for $Ca_9NaZn_{1-x}Mn_x(PO_4)_7$ (x = 0.05, 0.1, 0.2, 0.4, 0.5) samples.

x	0.05	0.1	0.2	0.4	0.5
**Data collection**					
Diffractometer	Rigaku SmartLab SE (3 kW sealed X-ray tube, D/teX Ultra 250 silicon strip detector, vertical type θ-θ geometry, HyPix-400 (2D HPAD) detector in the 2θ range between 3° and 110° with a step interval 0.02°)				
Radiation/Wavelength (k, Å)	CuK_α/1.5418 Å				
2θ range (°)	3-80				
Step scan (2θ)	0.02				
Number of points	3851				
I_{max}	20732	21243	19879	20407	20714
**Refinement**					
Background function	Legendre polynomials, 15 terms				
R/R_w for Bragg reflections, %	4.62/6.12	5.07/6.66	4.24/5.63	4.17/5.52	4.48/5.95
R_P, R_{wP}, R_{exp}, %	2.99/3.74/3.54	3.44/4.15/3.58	2.84/3.42/3.66	2.52/3.01/3.70	3.09/3.93/3.13
Goodness of fit (ChiQ)	1.73	1.86	1.65	1.49	1.60

Table S2. Atomic coordinates, isotropic atomic displacement parameters (Å²) and site-occupancy factors (SOFs) in $Ca_9NaZn_{0.5}Mn_{0.5}(PO_4)_7$.

Atom	*x*	*y*	*z*	*Uiso*	*SOF*
*M*1	0.7261(5)	0.8530(6)	0.4343(1)	0.0057	1 Ca
*M*2	0.6214(5)	0.8207(7)	0.2338(1)	0.0053	1 Ca
*M*3	0.1208(5)	0.2681(3)	0.3279(1)	0.0018	1 Ca
*M*4	0	0	0.1862(3)	0.0041	1 Na
*M*5	0	0	0.0036(1)	0.0151	0.5 Zn + 0.5 Mn
P1	0	0	0.2684(2)	0.0078	1
P2	0.6929(6)	0.8676(8)	0.1375(2)	0.0146	1
P3	0.6522(7)	0.8493(8)	0.0346(2)	0.0059	1
O1	0	0	0.3099(2)	0.0033	1
O2	0.0098(3)	0.8666(9)	0.2469(4)	0.0033	1
O3	0.7417(1)	0.9211(1)	0.1764(2)	0.0033	1
O4	0.7676(7)	0.7808(6)	0.1250(3)	0.0033	1
O5	0.7291(7)	0.0074(6)	0.1135(3)	0.0033	1
O6	0.5248(7)	0.7683(8)	0.1312(4)	0.0033	1
O7	0.6051(3)	0.9589(4)	0.0443(3)	0.0033	1
O8	0.5717(4)	0.6948(5)	0.0541(4)	0.0033	1
O9	0.8137(1)	0.9099(9)	0.0431(4)	0.0033	1
O10	0.6262(1)	0.8230(4)	−0.0062(2)	0.0033	1

Table S3. Atomic coordinates, isotropic atomic displacement parameters (Å^2) and site-occupancy factors (SOFs) in $Ca_9NaZn_{0.6}Mn_{0.4}(PO_4)_7$.

Atom	*x*	*y*	*z*	*Uiso*	*SOF*
*M*1	0.7274(4)	0.8548(5)	0.4345(1)	0.0057	1 Ca
*M*2	0.6195(4)	0.8220(6)	0.2340(1)	0.0053	1 Ca
*M*3	0.1204(4)	0.2684(3)	0.3278(1)	0.0018	1 Ca
*M*4	0	0	0.1882(2)	0.0041	1 Na
*M*5	0	0	0.0034(1)	0.0151	0.6 Zn + 0.4 Mn
P1	0	0	0.2677(2)	0.0078	1
P2	0.6891(5)	0.8608(7)	0.1377(2)	0.0146	1
P3	0.6510(6)	0.8465(7)	0.0340(2)	0.0059	1
O1	0	0	0.3092(2)	0.0033	1
O2	−0.1442(8)	−0.0085(2)	0.2524(3)	0.0033	1
O3	0.7485(1)	0.9249(8)	0.1748(2)	0.0033	1
O4	0.7671(5)	0.7758(4)	0.1249(3)	0.0033	1
O5	0.7320(4)	0.0040(3)	0.1141(3)	0.0033	1
O6	0.5205(7)	0.7647(6)	0.1337(3)	0.0033	1
O7	0.6093(2)	0.9636(1)	0.0465(3)	0.0033	1
O8	0.5743(3)	0.6945(2)	0.0547(3)	0.0033	1
*O*9	0.8209(1)	0.9206(5)	0.0425(3)	0.0033	1
*O*10	0.6234(9)	0.8213(3)	−0.0063(2)	0.0033	1

Table S4. Atomic coordinates, isotropic atomic displacement parameters (Å^2) and site-occupancy factors (SOFs) in $Ca_9NaZn_{0.8}Mn_{0.2}(PO_4)_7$.

Atom	*x*	*y*	*z*	*Uiso*	*SOF*
*M*1	0.7276(4)	0.8555(5)	0.4343(1)	0.0057	1 Ca
*M*2	0.6194(4)	0.8203(6)	0.2337(1)	0.0053	1 Ca
*M*3	0.1193(4)	0.2678(3)	0.3281(1)	0.0018	1 Ca
*M*4	0	0	0.1876(2)	0.0041	1 Na
*M*5	0	0	0.0030(1)	0.0206	0.8 Zn + 0.2 Mn
P1	0	0	0.2678(2)	0.0151	1
P2	0.6908(5)	0.8636(7)	0.1374(2)	0.0206	1
P3	0.6537(6)	0.8485(7)	0.0337(2)	0.0001	1
O1	0	0	0.3092(2)	0.0033	1
O2	−0.1409(9)	−0.0059(2)	0.2515(3)	0.0033	1
O3	0.7492(1)	0.9225(9)	0.1750(2)	0.0033	1
O4	0.7691(5)	0.7777(4)	0.1250(3)	0.0033	1
O5	0.7321(4)	0.0033(3)	0.1144(3)	0.0033	1
O6	0.5234(7)	0.7633(5)	0.1328(3)	0.0033	1
O7	0.6063(2)	0.9641(2)	0.0462(3)	0.0033	1
O8	0.5743(3)	0.6928(2)	0.0555(3)	0.0033	1
O9	0.8223(1)	0.9207(5)	0.0432(3)	0.0033	1
O10	0.6237(9)	0.8237(3)	-0.0066(2)	0.0033	1

Table S5. Atomic coordinates, isotropic atomic displacement parameters (Å^2) and site-occupancy factors (SOFs) in $Ca_9NaZn_{0.9}Mn_{0.1}(PO_4)_7$.

Atom	*x*	*y*	*z*	*Uiso*	*SOF*
*M*1	0.7252(5)	0.8542(6)	0.4344(1)	0.0057	1 Ca
*M*2	0.6199(5)	0.8199(7)	0.2339(1)	0.0053	1 Ca
*M*3	0.1193(5)	0.2682(4)	0.3282(1)	0.0018	1 Ca
*M*4	0	0	0.1877(3)	0.0041	1 Na
*M*5	0	0	0.0033(1)	0.0249	0.9 Zn + 0.1 Mn
P1	0	0	0.2680(2)	0.0087	1
P2	0.6910(6)	0.8652(9)	0.1379(2)	0.0216	1
P3	0.6519(7)	0.8470(9)	0.0344(2)	0.0033	1
O1	0	0	0.3094(3)	0.0033	1
O2	−0.1404(1)	−0.0065(4)	0.2500(4)	0.0033	1
O3	0.7534(1)	0.9212(1)	0.1752(3)	0.0033	1

O4	0.7662(8)	0.7735(7)	0.1251(4)	0.0033	1
O5	0.7338(8)	0.0051(5)	0.1145(3)	0.0033	1
O6	0.5249(9)	0.7643(9)	0.1329(4)	0.0033	1
O7	0.6056(4)	0.9653(4)	0.0465(4)	0.0033	1
O8	0.5689(5)	0.6988(4)	0.0555(4)	0.0033	1
O9	0.8152(2)	0.9169(8)	0.0435(4)	0.0033	1
O10	0.6264(1)	0.8255(5)	−0.0061(2)	0.0033	1

Table S6. Atomic coordinates, isotropic atomic displacement parameters (Å^2) and site-occupancy factors (SOFs) in $Ca_9NaZn_{0.95}Mn_{0.05}(PO_4)_7$.

Atom	*x*	*y*	*z*	*Uiso*	*SOF*
*M*1	0.7272(5)	0.8564(6)	0.4336(1)	0.0057	1 Ca
*M*2	0.6202(5)	0.8219(7)	0.2330(1)	0.0053	1 Ca
*M*3	0.1200(5)	0.2683(3)	0.3274(1)	0.0018	1 Ca
*M*4	0	0	0.1881(3)	0.0041	1 Na
*M*5	0	0	0.0027(1)	0.0214	0.95 Zn + 0.05 Mn
P1	0	0	0.2667(2)	0.0171	1
P2	0.6901(5)	0.8631(9)	0.1369(2)	0.0204	1
P3	0.6519(7)	0.8490(8)	0.0329(2)	0.0085	1
O1	0	0	0.3081(3)	0.0033	1
O2	−0.1445(1)	−0.0106(4)	0.2511(4)	0.0033	1
O3	0.7536(1)	0.9256(1)	0.1741(2)	0.0033	1
O4	0.7656(7)	0.7733(6)	0.1249(3)	0.0033	1
O5	0.7338(7)	0.0016(5)	0.1142(3)	0.0033	1
O6	0.5228(8)	0.7658(8)	0.1336(4)	0.0033	1
O7	0.6067(4)	0.9653(3)	0.0459(4)	0.0033	1
O8	0.5707(5)	0.6975(4)	0.0544(4)	0.0033	1
O9	0.8200(1)	0.9192(7)	0.0436(4)	0.0033	1
O10	0.6230(1)	0.8202(5)	0.9946(3)	0.0033	1

Table S7. Selected bond length in the $Ca_9NaZn_{1-x}Mn_x(PO_4)_7$ solid solutions.

		0.05	0.1	0.2	0.4	0.5
*M*1	O2	2.512(7)	2.497(7)	2.494(6)	2.487(6)	2.551(9)
	O4	2.903(8)	2.886(9)	2.924(6)	2.895(6)	2.912(8)
	O5	2.600(3)	2.593(3)	2.587(1)	2.586(1)	2.558(3)
	O6	2.492(4)	2.451(4)	2.434(1)	2.465(2)	2.449(4)
	O6	2.503(2)	2.522(2)	2.509(9)	2.494(1)	2.469(2)
	O7	2.443(4)	2.448(5)	2.461(1)	2.481(1)	2.541(2)
	O8	2.231(6)	2.225(6)	2.189(3)	2.224(3)	2.238(5)
	O10	2.445(2)	2.376(9)	2.382(9)	2.383(9)	2.383(9)
*M*2	O2	2.272(1)	2.298(1)	2.317(8)	2.289(8)	2.236(7)
	O3	2.520(9)	2.510(2)	2.498(8)	2.513(8)	2.427(8)
	O4	2.505(5)	2.489(8)	2.496(4)	2.476(4)	2.525(5)
	O5	2.323(4)	2.280(4)	2.301(3)	2.292(3)	2.267(5)
	O7	2.540(5)	2.534(6)	2.541(4)	2.537(3)	2.582(6)
	O8	2.795(8)	2.837(8)	2.748(6)	2.745(6)	2.804(8)
	O9	2.390(3)	2.396(4)	2.385(1)	2.406(1)	2.412(2)
	O9	2.424(9)	2.451(2)	2.441(7)	2.453(7)	2.477(5)
*M*3	O2	3.140(5)	3.222(5)	3.169(2)	3.115(2)	3.308(4)
	O3	2.580(8)	2.622(9)	2.597(8)	2.571(8)	2.606(9)
	O4	2.469(8)	2.483(1)	2.504(8)	2.487(8)	2.533(8)
	O5	2.418(3)	2.404(4)	2.417(2)	2.413(2)	2.416(3)
	O7	2.410(6)	2.402(6)	2.402(3)	2.435(3)	2.351(3)
	O8	2.616(5)	2.628(5)	2.609(2)	2.606(2)	2.588(5)
	O10	2.453(4)	2.494(4)	2.479(2)	2.467(2)	2.492(4)
	O10	2.566(7)	2.560(7)	2.547(4)	2.558(5)	2.570(6)
	O1	2.514(4)	2.508(5)	2.507(4)	2.508(4)	2.505(3)
*M*4	O2	2.746(6)	2.712(6)	2.767(2)	2.788(2)	2.675(6)

	O2	2.746(6)	2.712(6)	2.767(2)	2.788(2)	2.675(7)
	O2	2.746(7)	2.712(7)	2.767(3)	2.788(3)	2.675(6)
	O3	2.325(9)	2.305(9)	2.350(8)	2.368(8)	2.407(9)
	O3	2.325(1)	2.305(1)	2.350(1)	2.368(1)	2.407(8)
	O3	2.325(8)	2.305(9)	2.350(7)	2.368(7)	2.407(1)
*M*5	O6	2.155(1)	2.201(2)	2.186(4)	2.154(5)	2.252(8)
	O6	2.155(8)	2.201(8)	2.186(9)	2.154(9)	2.252(1)
	O6	2.155(1)	2.201(2)	2.186(9)	2.154(9)	2.252(1)
	O9	2.217(3)	2.229(3)	2.183(1)	2.166(1)	2.227(7)
	O9	2.217(6)	2.229(6)	2.183(3)	2.166(3)	2.227(2)
	O9	2.217(3)	2.229(3)	2.183(1)	2.166(1)	2.227(2)
P1	O1	1.535(3)	1.535(3)	1.534(1)	1.539(1)	1.542(1)
	O2	1.556(8)	1.570(9)	1.553(1)	1.558(1)	1.645(3)
	O2	1.556(1)	1.570(1)	1.553(7)	1.558(9)	1.645(9)
	O2	1.556(3)	1.570(3)	1.553(8)	1.558(7)	1.645(1)
P2	O3	1.526(1)	1.514(3)	1.520(1)	1.519(9)	1.540(1)
	O4	1.549(2)	1.572(4)	1.544(9)	1.537(9)	1.525(2)
	O5	1.523(6)	1.551(7)	1.545(4)	1.582(4)	1.580(7)
	O6	1.511(8)	1.512(9)	1.521(8)	1.524(8)	1.537(8)
P3	O7	1.570(9)	1.587(2)	1.574(7)	1.548(6)	1.489(9)
	O8	1.576(5)	1.545(5)	1.614(2)	1.565(2)	1.567(5)
	O9	1.565(3)	1.507(3)	1.558(1)	1.560(1)	1.500(2)
	O10	1.451(3)	1.521(1)	1.522(1)	1.521(9)	1.540(1)

Table S8. CIE coordinates for $Ca_9NaZn_{1-x}Mn_x(PO_4)_7$ solid solutions.

**Mn^{2+} concentration**	***x***	***y***
0.1	0.5020	0.4841
0.2	0.5080	0.4801
0.4	0.6705	0.3265
0.5	0.6789	0.3186
0.7	0.6847	0.3132
1.00	0.6897	0.3083
